# Chemical exposures and suspected impact on Gulf War Veterans

**DOI:** 10.1186/s40779-023-00449-9

**Published:** 2023-03-08

**Authors:** Rami Elhaj, Joseph M. Reynolds

**Affiliations:** Center for Cancer Biology, Immunology and Infection, Chicago Medical School, Rosalind Franklin University of Medicine and Science, North Chicago, IL 60064 USA

**Keywords:** Gulf War Illness (GWI), Sarin, Neuroinflammation, Organophosphate (OP)

## Abstract

Gulf War Illness (GWI) encompass a spectrum of maladies specific to troops deployed during the Persian Gulf War (1990–1991). There are several hypothesized factors believed to contribute to GWI, including (but not limited to) exposures to chemical agents and a foreign environment (e.g., dust, pollens, insects, and microbes). Moreover, the inherent stress associated with deployment and combat has been associated with GWI. While the etiology of GWI remains uncertain, several studies have provided strong evidence that chemical exposures, especially neurotoxicants, may be underlying factors for the development of GWI. This mini style perspective article will focus on some of the major evidence linking chemical exposures to GWI development and persistence decades after exposure.

## Background

Gulf War Illness (GWI) is a chronic multi-symptom disorder that severely impacted the health of nearly 300,000 veterans; roughly 4 out 10 of whom were deployed to the Persian Gulf during Operation Desert Shield and Desert Storm in 1990–1991. The symptomology that defines GWI includes chronic pain, fatigue, mood and memory impairments, lung diseases, gastrointestinal (GI) disturbances, and skin lesions [1]. Moreover, GWI veterans commonly were exposed to distressed conditions, contaminated environments, and possible chemical warfare agents. To deal with the regions’ pervasive insect and rodent populations, veterans often used and likely overused topical pesticides. Some of these pesticides included methyl carbamates, organophosphates (OPs), pyrethroids, and chlorinated hydrocarbons [2]. In Kuwait and Iraq, Gulf War Veterans (GWVs) were exposed to combustion products of more than 750 oil well fires [3]. In Khamisiyah, Iraq, GWVs were also possibly exposed to the byproducts of destroyed enemy munitions that included sarin and cyclosarin rockets [1–3]. Furthermore, to protect against possible warfare, troops were given tablets of pyridostigmine bromide (PB) and directed to take these pills whenever an attack was believed to be imminent [2].

The diagnosis of GWI using the Kansas definition requires symptomology in at least categories, including pain, sleep, cognition, respiratory, GI, and skin [4, 5]. The most common parallel of GWI to the general population is chronic sickness behavior, which can persist in similar symptomatic categories [6]. While chronic sickness behavior is not uncommon, the number of individuals affected remains difficult to decipher. However, chronic sickness behavior does share additional features with GWI, starting with the theory that symptoms may result from immune system dysregulation and/or neuroinflammation [7]. This article will discuss some of the potential long-term effects of different exposures, which may also share similarities with the general population. For example, chronic pesticide exposure has been linked to a broad spectrum of conditions, including nervous system perturbations [8]. Moreover, while the impact of stress on long-term health has been well documented in the past, the effects of stress on immunity are starting to be better understood [9].

This article aims to highlight some of the major chemical exposure agents associated with GWI, the theorized mechanisms of action, and their organ-specific and holistic effects that lead to GWI symptomology. While this article focuses on chemical agents, other factors such as stress are believed to contribute to GWI as well. The information in this article was obtained from different scientific databases and search engines such as PubMed, ScienceDirect, and Google Scholar. Keywords used and compounded included: “Gulf War Illness”, “Gulf War Veterans Illness”, “Persian War”, “organophosphates”, “pyridostigmine bromide”, “Sarin gas”, “Persian Gulf War”, “Neuroinflammation”, and “Neuroimmune”. The literature reviewed included peer-reviewed journal articles, and public and government agencies reports (e.g., United States Department of Veterans Affairs). Animal model studies were also included in the search to cover the applicability of the results and implications for future research and treatments.

## GWI and exposure

### Etiology of GWI

There are several hypothesized triggers for the development of GWI. Although the identification of a single etiological agent has remained elusive, most of the scientific literature reviewed in this article commonly suggest exposures to a combination of OP, carbamates, PB, and other pesticides that are causally associated with GWI and the neurological dysfunction exhibited by some GWVs [10]. A summary of some of the more prominent chemicals used to model suspected Gulf War (GW) exposure agents and their associated symptomology is presented in Table 1. Many of the research studies focus on the effects of GW chemical exposure agents on cellular, biochemical, physiological, and neuropsychological parameters; however, it is now apparent that modeling such exposures and their devastating effects decades later has certain limitations. Moreover, most of these exposure agents are used in rodent models, which have several limitations and can fail to account for parameters such as appropriate physiological dosing, aging, sex, comorbid conditions including obesity, and geographical environment among others.

**Table 1:** Chemical agents and surrogates commonly employed to model Gulf War era exposures

Author	Model	Agent of exposure	Type of chemical	Duration of exposure/concentration	Target system/organ studied	Symptomatic model targets	Results
Koo et al. [11]	Rat	CORT DFP	CORT – simulate organic stress reaction DFP – OP; sarin nerve agent surrogate	CORT in drinking water (200 mg/L in 0.6% EtOH) for 4 d, followed by a single injection of DFP (1.5 mg/kg, i.p.)	Evaluation of cerebral cortex	Neuroinflammation, cognitive impairment, sickness behavior	CORT exacerbates the neuroinflammatory response of DFP
Miller et al. [12]	Mouse	CPO DFP PHY CORT	CPO, PHY, DFP as surrogates for AChEIs	CPO (8 mg/kg), DFP (4 mg/kg), or PHY (0.5 mg/kg) was administered in the morning and returned to their home cage. CORT was given in the drinking water (400 mg/L in 1.2% EtOH) for 4 d prior to AChEI or vehicle exposure	Whole brain tissue	Neuroinflammation and general GWI symptomology	CORT blunts the impact of irreversible AChEI on AChE in the brain while exacerbating DFP and CPO neuroinflammatory response
O’Callaghan et al. [13]	Mouse	CORT DFP DEET	DEET is a common insecticide that was possibly overused in the GW	Mice were dosed once per day for 14 d with PB [2 mg/(kg·d), s.c.] and DEET [30 mg/(kg·d), s.c]. On days 8 – 15, mice received CORT in the drinking water (200 mg/L in 1.2% ethanol, EtOH). Finally, on day 15, mice were treated with a single injection of either DFP (4 mg/kg, i.p.) or saline	Frontal cortex Hippocampus Striatum Hypothalamus Olfactory bulbs Cerebellum	Neuroinflammation and sickness behavior	Mice exposed to DFP and CORT had a 300-fold exacerbated neuroinflammatory response compared to DFP alone
Murray et al. [14]	Mouse	PB CPF DEET	GW-related exposures to model prophylaxis, insecticides, and insect repellent	Mice were exposed daily for 2 weeks followed by RNA sequencing analysis of hippocampal tissue	Hippocampus	Neuronal health and cognition	Exposures resulted in enhanced gene expression profiles related to inflammation while decreasing expression of genes positively regulating neuronal health
Michalovicz et al. [15]	Mouse	Propranolol	Beta blocker	20 mg/kg in saline with CORT (200 mg/L in drinking water) or after exposure to CORT	Whole brain tissue	Neuroinflammation	Propranolol reduced mRNA expression of inflammatory cytokines such as TNF-α, CCL2, IL-1β in both the hippocampus and the cortex in the GWI model compared to normal healthy mice
Zakirova et al. [16]	Mouse	PB Permethrin	Nerve agent prophylaxis and pesticide	Daily injections for 10 d followed by cognitive and immunohistochemical staining at day 18 and at 5 months	Hippocampus Cerebral cortex	Cognitive impairment and astrogliosis	Exposure agents had little to no effect in the short term (18 d). At 5 months, treated animals exhibited cognitive impairments and astrogliosis
Patterson et al. [17]	Mouse	DFP CORT	Model for OP exposure and stress	CORT (200 mg/L) in drinking water for 7 d followed by DFP (1 mg/kg) on day 8	Intestinal tissue	Gastrointestinal dysfunction and neuroinflammation	Exposure to DFP reduces the expression of the intestinal tight junctions occludin
Jang et al. [18]	Mouse	CPF	Metabolite of CPO	Chronic treatment: mice were injected with 3 mg/kg of CPF for 14 d. Single dose treatment: mice were injected with 3 mg/kg of CPF	Whole brain Plasma	Skin toxicity and inflammation	CPF increased the levels of ROS in a human skin keratinocyte cell line (HaCaT)

*CORT* Corticosterone, *DFP* Diisopropyl fluorophosphate, *CPO* Chlorpyrifos oxon, *PHY* Physostigmine, *DEET* N,N-diethyl-meta-toluamide, *CPF* Chlorpyrifos, *PB* Pyridostigmine bromide, *AChE* Acetylcholine esterase, *AChEI* Acetylcholine esterase inhibitor, *GWI* Gulf War Illness, *GW* Gulf War, *OP* Organophosphate, *IL* Interleukin, *TNF-α* Tumor necrosis factor-alpha, *CCL2* CC Chemokine ligand 2, *EtOH* Ethanol, *ROS* Reactive oxygen species

### Inflammation as an underlying factor for GWI development and pathogenesis

To better understand the pathophysiology of GWI, one case–control observational study focused on defining the biomarkers that trigger the symptomology of this disease. Eleven GWI-associated blood biomarkers were identified, two of which were of particular interest because of their well-established role in inducing an inflammatory response: interleukin (IL)-6 and C-reactive protein (CRP) [1]. This study demonstrated that veterans with GWI symptoms exhibit a higher (2.5-fold) blood concentration of CRP as well as a significant increase in IL-6 protein compared to asymptomatic veterans. Thus, a positive correlation between IL-6 and CRP was established and these results further suggest that IL-6 may be one of the critical cytokines in driving the GWI inflammatory response [1]. Indeed, a mouse model of stress and OP exposure demonstrated substantial neuroinflammation in the brain with *Il6* being one of the most overrepresented cytokine genes [13]. The marker CRP is also commonly used in primary health care clinics to monitor many types of inflammatory diseases, autoimmune diseases and infections. Thus, IL-6 and CRP levels could potentially serve future utility as target biomarkers to manage GWI, monitor progression or remission post-treatment, and as a tool for early diagnosis of the disease. However, future studies with larger sample sizes are necessary to determine the utility of using these markers in this manner.

O’Donovan et al. [19] pioneered a study to investigate the relationship between inflammatory markers and hippocampal volume in the context of posttraumatic stress disorder. Even though the blood–brain barrier (BBB) provides sufficient protection to the brain from circulating solutes, peripheral inflammatory cytokines can still influence the brain by different mechanisms. For example, perivascular macrophages can interact with brain endothelial cells when the BBB structural integrity is compromised due to a direct insult or sepsis [19]. Thus, cytokines can provide a useful index to assess neuroinflammation. Higher levels of the inflammatory cytokine soluble receptor II for tumor necrosis factor (sTNF-RII) were correlated to reductions in overall hippocampal volume in a sample of GWV patients. This is partly due to the ability of neuroinflammation to inhibit hippocampal neurogenesis while promoting neuronal death, which could have the potential of drastically and adversely impacting memory and learning [19]. Further supporting work used sophisticated imaging techniques on GWVs with suspected sarin exposure and also demonstrated reduced hippocampal volume in these veterans [20]. Furthermore, because most of the GWI biomarkers found thus far are associated with inflammation and the inflammatory signal cascade, and inflammation is associated with practically all symptoms of GWI, it has been hypothesized that chronic inflammation is the underlying cause of this disease [1]. The idea of targeting inflammation, especially neuroinflammation, in GWI is not new; however, studies such as these highlight major reasons for doing so even for future therapeutic improvements to less obvious conditions such as cognition deficits.

### Arguments for acetylcholine esterase (AChE) dependent vs. independent mechanisms of action

During deployment, GWVs were potentially exposed to a variety of different acetylcholine esterase inhibitor (AChEI) toxicants including insecticides (such as carbamates and chlorinated hydrocarbons), OP, PB (an anti-nerve agent prophylactic), and possibly sarin and cyclosarin nerve gases. There is a strong correlation between these AChEI agents and the development of GWI [2]; however, there is still a debate on what could be the most plausible mechanisms of action. The impact of the reversible AChEI PB in particular has remained controversial (as reviewed in [21]). Although PB consumption has been linked to GWI, these findings are difficult to reconcile with the relatively safe use of PB and other AChEIs as therapeutics for autoimmune and neurodegenerative disease.

Acute toxic exposure to AChEIs has a predictable systemic effect that depends on overactivity of acetylcholine nicotinic and muscarinic receptors. These signs and symptoms include diarrhea, urination, miosis, bronchospasm, bradycardia/bradypnea, excitation of skeletal muscles and central nervous system, lacrimation, salivation, and sweating [22]. However, the long-lasting symptoms of GWI including mood disorders, cognitive and memory impairment, pain, similarity to adaptive sickness behavior response, and the absence of acute AChEI toxicity have suggested that GWI is related to an underlying chronic neuroimmune/neuroinflammatory disorder rather than an acute response to the direct inhibition of AChE-receptors [21]. Conversely, a recent study demonstrated a correlation between suspected low-level OP exposure, a specific polymorphism in the paraoxonase (*PON1*) gene, and the development of GWI [23]. *PON1* hydrolyzes and inactivated several substrates, including sarin. Therefore, altered activity of *PON1* at the genetic level may have increased susceptibility to GWI in certain individuals [23]. There are additional layers of complexity in terms of modeling OP exposure, mainly in that there exist other targets outside of AChE including several different enzymes and receptors (as reviewed in [24]). Further complicating matters is that all OP agents do not act the same and different targets will likely lead to different clinical symptoms [24]. Thus, it remains possible that the main underlying mechanism of action for individuals currently suffering from GWI is AChE independent as some studies have suggested; however, the identification of other OP targets that could also contribute to GWI pathogenesis remains mostly unexplored.

### Stress exacerbates the neuroinflammatory effects of chemical agents

GWVs experienced a tremendous amount of psychological stress as a result of deployment and combat which may have also exacerbated the effects of certain chemical exposures. GWI research commonly incorporates corticosterone (CORT) to mimic the systemic effects of physiological stress during deployment. Neuroinflammation has been linked to GWI previously [25] and remains one of the most prominent theorized explanations for the persistence of GWI even decades after exposure. In a GWI rat model, Koo et al. [11] evaluated neuroinflammation using a high-order diffusion magnetic resonance imaging (MRI) to detect underlying structural and connectivity changes between brain cells. After exposing these subjects to CORT and diisopropyl fluorophosphate (DFP, an organophosphorus surrogate for sarin), quantitative PCR results and a high order diffusion MRI showed evidence for exacerbated brain-wide neuroinflammation and inflammatory cytokine gene expression. Further supporting evidence demonstrated that CORT blunts the impact of irreversible AChEI on AChE in the brain while also exacerbating the neuroinflammatory response to DFP and chlorpyrifos oxon (CPO) [12]. This finding also suggested the possibility of an AChE-independent mechanism for AChEI-related neuroinflammation. Likewise, certain GW relevant exposures have also demonstrated the potential to initiate neuroinflammation without the manifestation of obvious inflammatory effects in the periphery [26], further indicating that neuroinflammation can be difficult to identify based solely on biomarker expression in the blood.

Another study led by O’Callaghan et al. [13] demonstrated that when mice were exposed to DFP plus CORT, the neuroinflammatory response was exacerbated up to 300-fold compared to exposure to DFP alone [13]. The mechanism of the neuroinflammatory augmentation of CORT is still not well understood and seems to be a paradoxical effect of an anti-inflammatory glucocorticoid. The unveiling of the impact of stress on neuroinflammation prompted a recent study led by Michalovicz et al. [15] to evaluate the potential effects of the drug propranolol, an anti-inflammatory β-adrenergic receptor blocker on neuroinflammation in a long-term mouse model of GWI. This model consisted of challenging animals with DFP followed by recurring CORT administration over a period of 5 weeks. Mice were then treated with propranolol 4 to 11 d prior to the administration of an inflammatory signal, lipopolysaccharide. They found that propranolol significantly reduced mRNA expression of inflammatory cytokines such as *Tnf*, *Ccl2*, and *Il1b* in both the hippocampus and the cortex in the GWI model compared to normal healthy mice [15]. The study is significant due to the use of a chronic, rather than acute, model that may better mimic what veterans with GWI currently experience and may help us to better investigate the long-term pathophysiology of the disease. This study also demonstrates that individuals were potentially primed for neuroinflammation through chemical exposure, which could further be exacerbated in periods of stress [15]. As such, an inflammatory signal unrelated to the initial exposure agent(s) may be the root cause of symptomatic appearance or relapse years after. Lastly, this study demonstrates the potential use of anti-neuroinflammatory drugs to treat GWI instead of treating individual symptoms while avoiding the suppression of a healthy immune response [15].

## Potential AChE independent mechanisms of action

### Gut dysbiosis

AChE expression is mostly limited to the nervous system, but other tissues are severely impacted by AChEIs as well. While these studies do not rule out the potential of AChE acting in a non-traditional manner, especially in the context of the enteric nervous system, it remains plausible that agents such as OPs impact other tissues through targeting different receptors or proteins [24]. One study led by Zhang et al. [27] explored the correlation between intestinal permeability in GWV and the development of chronic GI symptoms such as abdominal pain and diarrhea. In this study, intestinal permeability was evaluated using the urinary lactulose/mannitol test. In a normal intestine, lactulose is only slightly absorbed and acts as a marker of paracellular permeability. Thus, when ingested, lactulose is mainly excreted in the stool. On this premise, increasing the urinary lactulose/mannitol ratio means that intestinal wall integrity is compromised and is thus leaking substances into the bloodstream. Furthermore, the level of intestinal permeability was correlated with the mean ratings of daily abdominal pain, frequency of bowel movements, and consistency of stools in GWV with reported chronic GI symptoms [27]. Approximately 39.7% (29/73) of the participants with chronic GI symptoms had significant intestinal hyperpermeability and had higher abdominal pain ratings, looser bowel movements, and greater stool frequency than those with normal intestinal permeability [27]. The importance of this study is that it identified a new diagnostic biomarker (i.e., lactulose/mannitol ratio) that could potentially help with identifying neurotoxin exposure as well as improving diagnostic criteria for specific subgroups of GWI.

Other recent work also suggests that gut dysbiosis as a result of GW chemical exposure can be an initiator of the pro-inflammatory effects of GWI [28]. Exposing a rodent to CORT followed by PB and Permethrin [28] or DFP [17] results in significant alteration of intestinal microbiome composition. Moreover, transformation of the microbiome composition is associated with the reduction of healthy gut bacteria, while the loss of healthy gut bacteria will drastically impact the expression of the tight junction proteins occludin (decreased) and claudin-2 (increased) [29]. The imbalance of essential junctional proteins can compromise the integrity of the intestinal wall leading to a “leaky gut”, intestinal content flow (including endotoxins) into the systemic circulation, and neuronal inflammation as suggested by the increased Toll-like receptor 4 (TLR4) trafficking and activation [28]. Furthermore, amplification of TLR4-dependent signaling cascades results in increased activation and local inflammation in the small intestine, indicating that gut dysbiosis is a plausible mechanism for GI disturbances in GWI patients.

Gut dysbiosis is strongly associated with the pathogenesis of many inflammation-related disorders such as inflammatory bowel disease, chronic fatigue syndromes, and chronic liver disease, all of which have symptoms that resemble GWI [29]. Additionally, the loss of the gut bacteria *Lactobacillus* and *Bifidobacterium* species due to GW relevant exposure resulted in a significant reduction of the bacterial metabolite butyric acid. Butyrate/butyric acid is a known immunosuppressant that augments the differentiation of naïve T cells into CD4^+^ T regulatory cell cells, which suppresses the development of the pro-inflammatory CD4^+^ T helper 17 cells in the gut [30]. Furthermore, when treating permethrin and PB-exposed mice with butyrate through oral gavage, the levels of GPR109A (a butyrate receptor on T regulatory cells) significantly increased. Also, the presence of butyrate in the gut led to the restoration of the tight junction protein levels (claudin-2 and occludin) [29].

Another study, led by Patterson et al. [17], investigated the effects of DFP exposure on intestinal epithelium. They concluded that DFP-exposed mice significantly reduced tight junction proteins (claudin-4 and occludin) and their corresponding mRNA expression in the large intestine. This study also found that the loss of IL-17, a pro-inflammatory cytokine with protective properties in the intestinal epithelium, can exacerbate tight junction reduction in an acute model of GWI [17]. Moreover, exposure to DFP has led to a significant decrease in the antimicrobial peptide (AMP) secreted by intestinal epithelial cells. The loss of AMP compromises the intestinal microbiome and may underlie to dysbiosis [17]. Collectively, these works implicate that gut decontamination of GWI patients coupled with restoring a healthy gut microbiome and introducing essential bacterial metabolites such as butyrate and intestinal cytokines such as IL-17 may attenuate certain symptoms with the caveat that additional research is a requirement before clinical testing can be justified.

### Axonal transport

Trafficking essential proteins, vesicles, and organelles such as mitochondria along the neuron is critical for the axonal outgrowth and function of a neuronal cell [31]. However, exposing cerebral cortical neurons to DFP caused an impairment of anterograde and retrograde transport of membrane-bound organelles, as seen in a time-lapse imaging technique [32]. Moreover, other studies confirmed that a similar impact of OP on axonal transport could be demonstrated in an in vivo rat model using manganese-enhanced magnetic resonance imaging (MEMRI) [33, 34]. Some experiments suggest that axonal transport deteriorates due to the ability of OPs to bind and modify some of the tyrosine residues in a human kinesin 3C motor domain and weaken the kinesin-microtubule interaction, which is vital for anterograde axonal transport [35, 36]. Another study supports the ability of OPs to destabilize tubulin polymerization by covalently binding to the tyrosine residues on tubulin and halting axonal transport [37]. OPs can also lead to the inhibition of tubulin acetylation and thus impair the movement of membrane-bound organelles along the axons of human and rodent neurons [38]. Lastly, impairments of axonal transport could be detected at OP concentrations that did not inhibit AChE activity, and using cholinergic antagonists did not slow down the rate of cellular injury [39]. These findings and imaging techniques may help better screen for axonal transport deficits and provide a better diagnosis of OP-related GWI.

### Oxidative stress and apoptotic neurodegeneration

Another possible non-AChE mechanism of action is the ability of multiple OPs to trigger apoptotic neurodegeneration by raising oxidative stress. Chlorpyrifos (CPF) increased the levels of reactive oxygen species (ROS) [40] in a human skin keratinocyte cell line (HaCaT) [18]. This was detected by a 2',7'-dichlorodihydrofluorescein diacetate assay that determines intracellular ROS production (e.g., hydrogen peroxide, hydroxyl radicals). Moreover, the decreased glutathione to oxidized glutathione ratio confirmed the effect of CPF on ROS production [18].

The increase in ROS can trigger an intrinsic pathway of programmed cell death (apoptosis) by increasing Ca^2+^ influx into the mitochondria and the initiation of a proteolytic pathway that accelerates the degradation of cellular components and cell membrane integrity [41]. Using a TUNEL assay, Jang et al. [18] were able to visualize an increase in apoptotic DNA cleavage caused directly by CPF compared to that in control cells. Further support for mitochondrial dysfunction in GWI was provided by another study demonstrating increased levels of mitochondrial DNA lesions and mitochondrial DNA copy number in blood samples from 21 individuals with GWI compared 7 control subjects [42]. Moreover, GW era exposures were investigated in a rodent model for potential alterations in the brain gene expression profiles of several oxidation, mitochondrial activity, and inflammation-related pathways [43]. This group found that pesticide exposure in combination with stress resulted in enhanced hippocampal expression of genes governing the positive regulation of oxidative stress, ROS, and mitochondrial activity. They also demonstrated that these elevated pathways were associated with pro-inflammatory gene expression, further indicating that oxidative stress and mitochondrial dysfunction may be associated with neuroinflammation.

In a recent study that investigated the association between pesticide exposure and Parkinson’s disease, human neuroblastoma cell line SH-SY5Y cells that were exposed to CPF showed a significant decrease in viability, an increase in ROS production, and upregulation of pyroptosis related proteins (e.g., caspase-1, IL-1β, and IL-18) [44]. Pyroptosis is a type of programmed cell death dependent on the inflammatory process of NLRP1/caspase-1 signal [44]. Even though these studies do not directly relate these processes to the development and progression of GWI, it sheds light on a possible explanation of how CPF and other OPs can cause neuroinflammation and degeneration in a GWVs. Future studies should attempt to find a direct relationship between the induction of pyroptosis and ROS production in a GWI model.

### Gene expression

New techniques in gene sequencing have paved the way to understanding the effects of neurotoxicants such as OPs on epigenetic programming using GWI models. A reduced representation bisulfite sequencing (RRBS) showed a significant increase in DNA methylation in the prefrontal cortex (PFC) and the hippocampus after exposing mice to CORT and DFP, which led to altered expressions of immune-related genes [45]. Moreover, exposure to DFP caused an enrichment of genes related to histone modification, thus increasing the level of chromatin accessibility and transcription as measured by the biomarker H3K27ac [45]. Some of these changes are associated with a discrepancy in M1 and M3 acetylcholine receptor expression and synaptic functions compared to controls. These findings are consistent with the cognitive and memory impairment seen in GWI attributed to changes in the PFC and hippocampal physiology [45, 46]. Epigenetic alterations observed in rodent studies are more difficult to link to chemical exposures in individuals with GWI, especially in the context of said exposures occurring decades in the past. However, a recent pilot study has uncovered global epigenetic alterations manifesting with GWI [47]. This group found that while global DNA methylation patterns remain unchanged, hundreds of mostly hypermethylated CpG sites were discovered in individuals with GWI. What remains to be determined though is whether these alterations can be linked to specific GW era exposures.

To continue to model the genetic alterations that occurs in those with GWI, Xu et al. [48] examined whole-genome RNA sequences in the PFC of 30 BXD recombinant inbred mouse strains with a combined treatment of CORT + DFP. This helped them identify ample numbers of differentially expressed genes (DEGs) among the 30 strains. A large portion of them participated in the immune system were upregulated by DFP + CORT [48]. The evidence of variable expression of genes across the BXD strains indicates that genomics plays a significant role in the response that CORT + DFP can elicit in mice [48]. This may help explain why we see the inconsistency of GWV’s susceptibility to toxicants and why we see different predominating symptoms among GWI sufferers. Furthermore, quantitative trait loci mapping identified loci of genes that were differentially expressed when exposed to the treatment of CORT + DFP only. One of significant interest is the gene *Adamts 9*, which can influence cytokine-related gene expression and correlate with the development of inflammation, cognitive aging, and chronic fatigue, which all can be seen in GWI [48].

## GWI animal models: benefits and limitations

### Possible benefits for GWI

Preclinical animal models of disease are a necessary first step in understanding pathophysiology as well as identifying potential therapeutic interventions. Thus, the development of GWI animal models represents a significant milestone, especially for those with the capacity to simulate GWI-like physiological responses following exposure of GW chemical toxins and other insults. Common agents include PB, permethrin, DFP, CORT, and DEET to mimic the nerve agent sarin, physiological stress, and pesticides, respectively [13]. Furthermore, these treatments are used to elicit a chronic or an episodic response to emulate sickness behavior and the chronic progression of GWI. Additionally, experiments using GWI animal models provide a vehicle to test the different hypotheses that gave rise to the constitution of symptoms and further test the possible treatments available to ameliorate the symptoms of GWI. A non-exhaustive list of some of the more prominent GWI models and associated findings is presented in Table 1.

Nonhuman primates are considered the best model to test OP intoxication because of their physiological, anatomical, and behavioral similarities with humans. However, due to the limitations with the availability of nonhuman primate facilities, this type of research is often conducted on rodents. Rats and mice are widely used in research for studying the acute and delayed consequences of OP exposure because of rapid lifespans, analogous molecules with humans, affordability, and small size, which helps experiments become more manageable [49]. Additional advantages to mouse models include the ability of scientists to more readily manipulate mouse DNA and investigate the effects of specific gene function and dysfunction in different conditions such as GWI.

Researchers use numerous laboratory techniques to investigate the pathophysiology of GWI in rodent animal models. For example, many use an electrochemiluminescence assay to measure the levels of the inflammatory biomarkers interferon-α, IL-1β, tumor necrosis factor-α, etc.. Others have employed ELISA-based methods to quantify human IL-6 and sTNF-RII protein [19]. These techniques help compare the levels of inflammation induced by GWI-relevant chemicals in comparison to control participants or exposures. In addition, many GWI researchers have used imaging techniques to visualize the effects of toxicant exposures on the brain’s function and structure. For example, 1.5-Tesla vision MRI scanners were used to acquire T1-weighted 3D volumetric images to quantify changes in hippocampal volumes [19].

Moreover, MEMRI was used to measure neuronal activity and visualize deficits in axonal transport [38]. Calcium and nitric oxide imaging and microscopy were used to visualize the effects of PB on GI motility [50]. This helped identify the pathophysiology of the GI component of GWI. Furthermore, using new technology such as the Illumina HiSeq, enabled researchers to rapidly and effectively identify mRNA sequences and differentiate gene expression between chemically exposed mice and controls.

### Limitations

Researchers face many limitations when designing an animal model for GWI because there is still speculation on the types of toxicants, levels of exposure, and the diverse presentations of the disease. Moreover, aging and co-morbid conditions are particularly difficult to model in rodents, which further contribute to challenges to creating universal models to represent all of GWI [3] Thus, mouse models can be easily over-interpreted. Furthermore, many of the symptoms that GWVs present with, including headaches, joint pain, and muscle pain, are difficult to assess in an animal model. Thus, it becomes challenging to interpret animal study data to define GWI in an animal model [3]. Another limitation is the disproportionate focus on male species to model the disease. This creates a disparity in diagnosing and treating women veterans with GWI [51].

Another major limitation of animal GWI models is the difficulty in balancing symptom appearance with the time needed for an effective study. The short life span of rodents is generally considered to be beneficial in terms of being able to monitor the progression of chronic disease as well as helping to identify markers of genetic susceptibility, inflammatory damage, and other factors involved in chronic disease regulation. However, some conditions or symptoms don’t develop until much later in a rodent’s life [52]. Thus, more studies should also be dedicated to studying the effects of chemical agent exposure across the lifespan of the test subject, despite the costs and/or labor associated with doing so. This is especially relevant for the current state of GWI as one epidemiological study has demonstrated that individuals with GWI exhibit higher rates of conditions associated with individuals much older, including cardiovascular disease and arthritis [53].

When studying GI inflammation in GWI, it is essential to consider unique behavioral patterns in nonhuman subjects that can impact the induction and progression of intestinal diseases [54]. For example, coprophagy in mice, the nocturnal re-ingestion of nutrients, can potentially affect intestinal health by influencing GI microbial populations [54]. Thus, the extrapolation of the effects of these behaviors on disease processes in rodents to humans has the potential to create inaccurate analyses or interpretations.

## Conclusions

The precise mechanism of developing GWI, and the link between chemical (and other) exposures and the chronic manifestation of the disease is still not fully agreed upon in the scientific and medical community. However, common patterns help unify many studies that can guide the development of diagnostic tools or even treatments. This article dives into the hypothesized pathophysiology of GWI that has caused thousands of veterans to suffer from a constellation of chronic symptoms such as pain, fatigue, mood and memory impairments, lung diseases, GI disturbances, and skin lesions [2]. Gut dysbiosis, axonal transport dysregulation, apoptotic neurodegeneration, oxidative stress, and gene dysregulation are some of the hypothesized mechanisms of action associated with AChEI-induced neuroinflammation and organ dysregulation. Furthermore, these studies have identified essential biomarkers such as lactulose/mannitol ratio, Adamts 9, IL-6 expression, and new imaging techniques such as MEMRI that can facilitate better screening and an earlier diagnosis of GWI. Many veterans report that health care providers fail to recognize GWI partially due to an unestablished biomarker that can point towards this disease [55]. For this reason, the management of patients with GWI or “medically unexplained” physical symptoms (MUS) costs twice as much in healthcare resources than patients with a more established diagnosis. One study reported that of people who met the criteria for a MUS condition, only 14% were previously diagnosed with it. Another study found that less than 50% of subjects were properly diagnosed with a MUS in an initial patient encounter. Furthermore, many patients with MUS/GWI are misdiagnosed as a mental health condition and thus were poorly managed [55].

By establishing common biomarkers and imaging patterns for GWI, healthcare providers will be more equipped to diagnose GWI in veterans properly as GWV have been experiencing several disabilities for a long time. The potential of providing earlier diagnoses or a diagnosis that could point towards some form of individualized therapy may provide for significant relief. Some of the studies highlighted in this article do imply possible treatments for GWI, such as anti-inflammatory medication and gut decontamination to combat potential effects of AChEI. However, the potential for treating other underlying mechanisms for GWI, such as altered epigenetic profiles or mitochondrial dysfunction, is more complicated and requires further research.

One limitation in some GWI studies is the lack of objective data on exact dosage and agents that GWVs were exposed to due to an overreliance on self-reporting. Further confounding variables include comorbities and potential impacts for other suspected GWI factors, including exposure to burn pits and oil well fires. There is also potential for more mechanical implications as a recent study demonstrated that GW exposure agents exacerbate mild traumatic brain injury [56]. Another limitation is the lack of consistency in symptoms among GWV that impedes establishing causality. In addition, many patients with symptoms that constitute a diagnostic criterion of GWI, did not know that their illnesses are service-connected thus could have underestimated the effects of GW exposures. Future experiments and studies should delve deeper into explaining AChE-independent mechanisms, which could result in better treatment methods for GWI.

## Data Availability

Not applicable.

## Abbreviations

AChE: Acetylcholin esterase
AChEI: Acetylcholin esterase inhibition
AMP: Antimicrobial peptide
BBB: Blood–brain barrier
CORT: Corticosterone
CPF: Chlorpyrifos
CPO: Chlorpyrifos oxon
CRP: C-reactive protein
DFP: Diisopropyl fluorophosphate
GI: Gastrointestinal
GWI: Gulf War Illness
GWVs: Gulf War Veterans
IL: Interleukin
MEMRI: Manganese-enhanced magnetic resonance imaging
MRI: Magnetic resonance imaging
MUS: “Medically unexplained” physical symptoms
OP: Organophosphate
PB: Pyridostigmine bromide
PFC: Prefrontal cortex
ROS: Reactive oxygen species
sTNF-RII: Soluble receptor II for tumor necrosis factor
